# Neurons and guidance cues in retinal vascular diseases

**DOI:** 10.18632/oncotarget.7413

**Published:** 2016-02-15

**Authors:** Ariel Wilson, Przemyslaw Sapieha

**Affiliations:** Department of Ophthalmology and Biochemistry and Molecular Medicine, Maisonneuve-Rosemont Hospital Research Centre, University of Montreal, Montreal, Quebec, Canada; and Department of Neurology-Neurosurgery, McGill University, Montreal, Quebec, Canada

**Keywords:** angiogenesis, retinopathy, diabetic, prematurity, vessels

Retinal vasculopathies such as diabetic retinopathy (DR), retinopathy of prematurity (ROP), and age-related macular degeneration (AMD) are the most common causes of vision loss in the industrialized world. According to the National Eye Institute, 7.7 million Americans are affected by DR, an additional 10 million have AMD, and of the 3.9 million infants born annually in the U.S., ∼15 000 are affected to some degree by ROP. In wet AMD, unchecked proliferation of choroidal vessels leads to photoreceptor compromise and vision loss. In ischemic retinopathies such as ROP and DR, degeneration of retinal vessels is followed by misguided pathological neovascularization. Recent studies on DR, ROP and AMD have revealed the paramount role for neurons and neuronal guidance cues in disease progression [1-6]. During retinal embryogenesis, coordinated interplay between neurons, blood vessels and immune cells is critical for proper retinal development. Although neurovascular and neuroimmune crosstalk shapes vascular development in the retina, it has received limited attention in disease etiology. A better understanding of this crosstalk may provide novel druggable targets to counter vasodegenerative and vasoproliferative eye disease. Here we briefly highlight new evidence suggesting that CNS neurons and guidance cues secreted by neurons such as retinal ganglion cells (RGCs) and photoreceptors have an inherent ability to influence vascular and immune responses in retinopathies.

Photoreceptors were likely the first neurons suggested to influence the progression of pathological retinal neovascularization. For instance, a negative correlation was drawn between incidences of retinitis pigmentosa, which causes photoreceptor degeneration, and proliferative DR [7]. Further investigation demonstrated that retinal neovascularization associated with long-standing diabetes mellitus spontaneously regressed with the onset of retinitis pigmentosa [8]. This observation held true in animal models where mice with genetically ablated photoreceptors failed to mount reactive retinal neovascularization in a model of oxygen-induced retinopathy (OIR), which mimics the vasodegenerative phase of ROP and loosely represents the vasoproliferative phases of ROP and DR [8]. These studies draw a link between neuronal energy demand and retinal neovascularization.

A mechanism via which central neurons communicate with their environment is through production of classical neuronal guidance cues such as semaphorins, netrins, and ephrins. These proteins are now widely regarded to have angiogenic and inflammatory functions, underscoring their pleiotropic nature. While these signaling proteins were initially thought to exclusively pattern the nervous system, it is now clear they also play a critical role in blood vessel development and influence the immune response during embryogenesis, and tissue homeostasis in adulthood. If discovered by an immunologist, they would have been classified as cytokines, by a vascular biologist, they would have been labeled angiomodulatory factors.

In addition to photoreceptors, RGCs have the propensity to significantly influence their retinal angiogenesis (either positively [3] or negatively [4]), and are directly apposed to the retinal vasculature that degenerates in DR and ROP. For example, in the mouse model of OIR, the guidance cue SEMA3A is secreted by hypoxic neurons, repelling regenerating vessels from the most ischemic regions of the retina [6]. Silencing *Sema3A* enhances physiological vascular regeneration of the hypoxic retina [6]. In non-proliferative DR, SEMA3A is produced by RGCs in hyperglycemic conditions and contributes to inner blood-retinal barrier disruption and consequent macular edema [1]. Therapeutic neutralization of SEMA3A reduces pathological retinal vascular permeability [1].

In addition to its influence on blood vessels, SEMA3A also affects immune cells such as mononuclear phagocytes (microglia/macrophages) that are central to the progression of proliferative retinopathies. While cytokine signatures responsible for retinal inflammation in DR and ROP are well characterized (notably IL-4, IL-6 and TNFα), the pro-inflammatory properties of neuronally expressed SEMA3A (and VEGF) in retinopathies highlight their worth as therapeutic targets. Hypoxic retinal neurons secrete SEMA3A and VEGF, attracting pro-angiogenic mononuclear phagocytes to sites of pathological neovascularization [2]. These mononuclear phagocytes infiltrate the retina, take on a microglial phenotype and actively partake in vascular degeneration and later pathological angiogenesis [2]. Therapeutic inhibition of SEMA3A (and VEGF) consequently reduces ischemia-driven retinal inflammation [2].

In counterpart, certain guidance cues are critical for maintenance of retinal homeostasis. Under physiological conditions, Netrin-1 polarizes retinal microglia towards a reparative phenotype [3]. In retinopathy, ischemia activates pathways of ER-Stress that degrade Netrin-1 and hence short circuit reparative neuroimmune communication. Degradation ofNetrin-1 by the IRE1α arm of ER stress impedes physiological vascular regeneration [3]. Furthermore, vasorepellent SEMA3F is expressed in healthy, avascular outer layers of mouse and human retinas, at the photoreceptor/retinal pigment epithelium (RPE) interface [5]. *SEMA3F* is significantly downregulated in the RPE layer of patients with AMD showing pathological choroidal neovascularization [5]. Reduction in levels of SEMA3F in the outer retina may therefore compromise a chemical barrier that maintains the photoreceptor layer avascular.

In sum, there is growing evidence that neuronal guidance cues actively partake in retinal vasculopathies such as ROP, DR and wet AMD. While current therapeutic strategies for these blinding diseases focus largely on blocking pathological angiogenesis through inhibition of VEGF, deleterious off-target effects are emerging due to the vaso- and neurotrophic properties of this factor. Pharmacological modulation of guidance cues such as semaphorins and netrins may offer alternative strategies to restore healthy functional retinal vasculature to ischemic zones of the retina and consequently reduce the destructive inflammation associated with these diseases.
